# A noncanonical heme oxygenase specific for the degradation of *c*-type heme

**DOI:** 10.1016/j.jbc.2021.100666

**Published:** 2021-04-17

**Authors:** Shuxin Li, Eta A. Isiorho, Victoria L. Owens, Patrick H. Donnan, Chidinma L. Odili, Steven O. Mansoorabadi

**Affiliations:** Department of Chemistry and Biochemistry, Auburn University, Auburn, Alabama, USA

**Keywords:** *c*-type heme, heme oxygenase, iron metabolism, peptide transport, structure–function, ABC, ATP-binding cassette, ABM, antibiotic biosynthesis monooxygenase, CHO, *c*-type heme oxygenase, HCP1, heme carrier protein 1, HO, heme oxygenase, HPLC, high-performance liquid chromatography, IPTG, isopropyl β-d-1-thiogalactopyranoside, LB, Luria–Bertani, MP-9, microperoxidase-9, MP-11, microperoxidase-11, MS/MS, tandem mass spectrometry, Opp, oligopeptide permease, PMP, pyridoxamine 5′-phosphate

## Abstract

Heme oxygenases (HOs) play a critical role in recouping iron from the labile heme pool. The acquisition and liberation of heme iron are especially important for the survival of pathogenic bacteria. All characterized HOs, including those belonging to the HugZ superfamily, preferentially cleave free *b*-type heme. Another common form of heme found in nature is *c*-type heme, which is covalently linked to proteinaceous cysteine residues. However, mechanisms for direct iron acquisition from the *c*-type heme pool are unknown. Here we identify a HugZ homolog from the oligopeptide permease (*opp*) gene cluster of *Paracoccus denitrificans* that lacks any observable reactivity with heme *b* and show that it instead rapidly degrades *c*-type hemopeptides. This *c*-type heme oxygenase catalyzes the oxidative cleavage of the model substrate microperoxidase-11 at the β- and/or δ-meso position(s), yielding the corresponding peptide-linked biliverdin, CO, and free iron. X-ray crystallographic analysis suggests that the switch in substrate specificity from *b*-to *c*-type heme involves loss of the N-terminal α/β domain and C-terminal loop containing the coordinating histidine residue characteristic of HugZ homologs, thereby accommodating a larger substrate that provides its own iron ligand. These structural features are also absent in certain heme utilization/storage proteins from human pathogens that exhibit low or no HO activity with free heme. This study thus expands the scope of known iron acquisition strategies to include direct oxidative cleavage of heme-containing proteolytic fragments of *c*-type cytochromes and helps to explain why certain oligopeptide permeases show specificity for the import of heme in addition to peptides.

Iron is an essential element for life. Organisms have therefore developed sophisticated machinery for obtaining, transporting, and storing iron (1). Iron acquisition is particularly important for bacterial pathogens to establish and maintain infections (2). Many pathogenic bacteria synthesize and secrete siderophores, high-affinity iron chelators that scavenge free ferric iron (3). The Fe(III)-siderophore complex is then imported into the cell, where the iron is released (*e.g.*, *via* reduction to the less tightly bound ferrous state) (4). However, in many hosts, including humans, iron is predominantly found complexed with protoporphyrin IX in the form of heme. Thus, bacteria also typically contain transport systems to acquire heme from the environment (5). A dedicated enzyme, heme oxygenase (HO), is then required to liberate the tightly bound iron from heme under aerobic or microaerophilic conditions (6).

In addition to serving as the substrate of HO, heme is utilized as a cofactor that facilitates its own oxidative cleavage (7). There are several distinct groups of HO, which differ in their structural folds and in the regiospecificity of their ring cleavage reactions (8). The first HO, HO-1, was isolated from rat microsomes in the late 1960s and is a monomeric α-only protein (9, 10, 11). HO-1 and most other canonical HOs from this group (such as HmuO from *Corynebacterium diphtheriae* and HemO from *Neisseria meningitidis*) cleave heme at the α-meso position, releasing ferrous iron and producing biliverdin IXα (α-biliverdin) and carbon monoxide as coproducts (Fig. 1) (12, 13). However, the HemO homolog PigA from *Pseudomonas aeruginosa* was shown to cleave heme at both the β- and δ-meso positions to yield a mixture of β- and δ-biliverdins (Fig. 1) (14).

**Figure 1:**
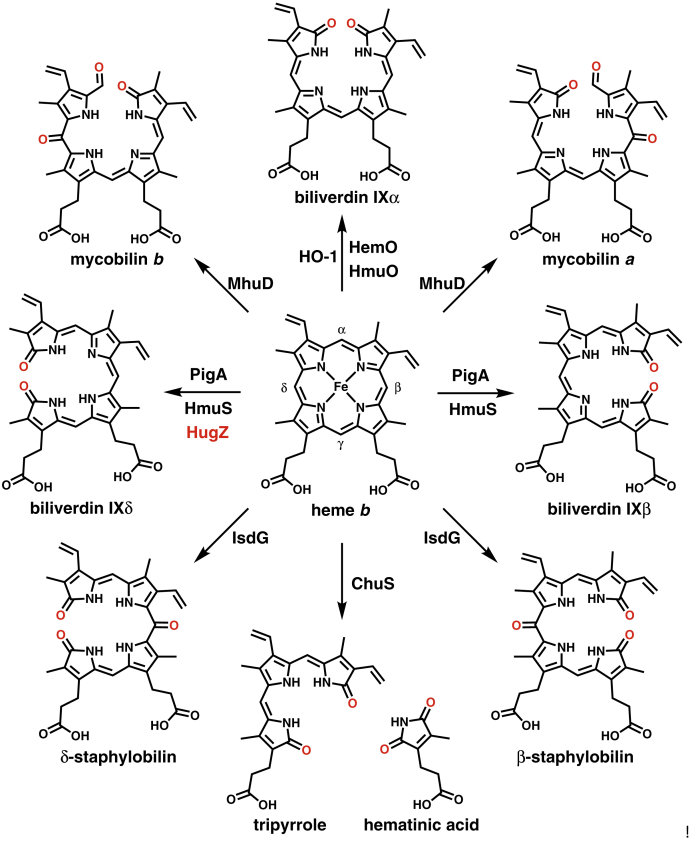
**Products of free *b*-type heme degradation generated by the various groups of heme oxygenase (HO)**.

The second group of HOs are structurally and functionally related to the iron-regulated surface determinants protein, IsdG, from *Staphylococcus aureus* (15). IsdG belongs to the antibiotic biosynthesis monooxygenase (ABM) superfamily and contains a ferredoxin-like fold (16). In contrast to canonical HOs, IsdG cleaves heme at the β- or δ-meso positions to form a mixture of β- and δ-staphylobilins and formaldehyde (Fig. 1) (17, 18). The structure of staphylobilin differs from that of the corresponding biliverdin by the presence of an oxo group at the unopened β- or δ-meso position. Another member of this group, MhuD from *Mycobacterium tuberculosis*, cleaves heme at the α-position; however, the meso carbon is retained in the product as a formyl group, yielding a mixture of mycobilins *a* and *b* (Fig. 1) (19). Mycobilins *a* and *b* also contain an oxo group at the β- and δ-meso position, respectively.

The third group of HOs contain tandem repeats of a motif named after its founding member, HemS (20). The HemS homolog HmuS from *Yersinia pseudotuberculosis* was found to convert heme to a mixture of β- and δ-biliverdins, analogous to the reaction catalyzed by PigA (Fig. 1) (21). In contrast, ChuS, a HemS homolog from *Escherichia coli* O157:H7, was shown to facilitate the peroxide-dependent conversion of heme to hematinic acid and a tripyrrole *via* ring cleavage at adjacent meso positions (*i.e.*, γ/β and/or γ/δ) (Fig. 1) (22).

The final group of HOs belong to the HugZ superfamily, as typified by the enzyme from *Helicobacter pylori*, which cleaves heme specifically at the δ-meso position and produces δ-biliverdin (Fig. 1) (23). Homologs of HugZ are abundant in Proteobacteria and belong to a larger superfamily of dimeric split β-barrel enzymes that include flavin (FMN/FAD)- and deazaflavin (F_420_)-dependent oxidoreductases (24). The two heme-binding sites of HugZ are located at the intermonomer interface (Fig. 2*A*) (25). Notably, the active site is framed by the N-terminal α/β-type domain, which interacts with both the α-meso edge of heme and the C-terminal loop containing a histidine residue that serves as the axial ligand to the heme substrate (Fig. 2*A*) (25).

**Figure 2:**
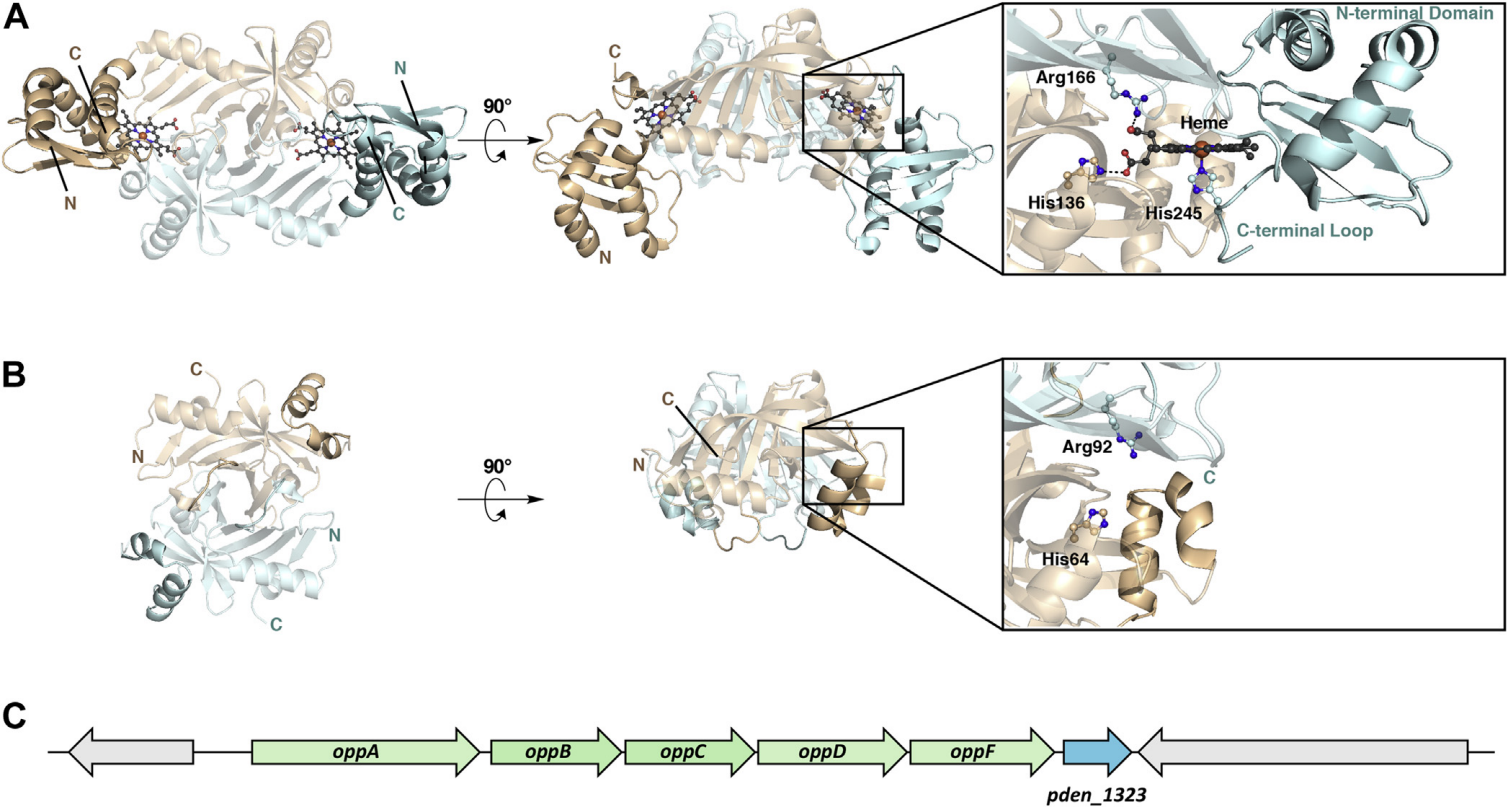
**Structural comparison of HugZ and Pden_1323.***A*, X-ray crystal structure of HugZ (PDB ID code: 3GAS) (25). The N-terminal α/β domain and C-terminal loop that are absent in the structure of Pden_1323 are *highlighted*. The heme substrate and several interacting active site residues (His136, Arg166, and His 245) are shown as ball-and-sticks. *B*, X-ray crystal structure of Pden_1323 (deposited PDB ID code: 6VNA). Dynamic regions showing varying degrees of disorder within the monomers of the asymmetric unit are *highlighted*. The His and Arg residues that interact with the carboxylates of heme are conserved in the structure of Pden_1323 and are shown as *ball-and-sticks* (His64 and Arg92). *C*, gene cluster showing the location of *pden_1323* within the oligopeptide permease (*opp*) operon.

Linking all known HOs across these structural and functional differences is a preferential degradation of free, *b*-type heme as their substrate (8). Yet in addition to *b*-type heme, one of the most prevalent forms of heme found in nature is *c*-type heme, wherein the vinyl groups of the porphyrin macrocycle are covalently linked *via* thioether bonds to proteinaceous cysteine residues from a conserved CXXCH motif (26). The terminal histidine residue of this *c*-type heme-binding motif serves as the axial ligand to the heme iron (26). HO-1 was previously shown to have some activity with heme *c* derivatives, although the catalytic efficiency was more than an order of magnitude less than with free heme (27, 28). Thus, specific mechanisms for direct iron acquisition from the *c*-type heme pool remain unknown.

*Paracoccus denitrificans* is a metabolically versatile Gram-negative alphaproteobacterium, capable of chemoautotrophic growth on hydrogen/thiosulfate and C_1_ compounds (*e.g.*, CO_2_, methanol, methylamines) or heterotrophic growth using oxygen (aerobic respiration) or nitrate/nitrite (denitrification) as terminal electron acceptors (29). In each of these metabolic pathways, *c*-type cytochromes (heme *c* binding proteins such as cytochrome *c*_550_, *bc*_1_, and *cd*_1_) play critical roles as components of the relevant electron transport chains (30).

The genome of *P. denitrificans* contains a cluster of genes that encode a homolog of the oligopeptide permease (Opp) system (Fig. 2*C*) (31). The Opp system is an ATP-binding cassette (ABC)-type transporter consisting of a membrane-associated substrate-binding lipoprotein (OppA), a hydrophobic transmembrane channel (OppBC), and two intracellular ATPase subunits (OppDF). OppA is a cluster C type substrate-binding protein, members of which have recently been shown to bind and facilitate the transport of heme in addition to short peptides (32, 33). Within the *opp* operon is a gene (*pden_1323*) annotated by the National Center for Biotechnology Information (NCBI) as a pyridoxamine 5′-phosphate (PMP) oxidase-related FMN-binding protein (Fig. 2*C*). PMP oxidases belong to the same superfamily as HugZ-like HOs, which motivated investigation of Pden_1323 as a HO (24).

Here, we show that Pden_1323 is an unusual HO that shows specificity for the degradation of *c*-type heme instead of *b*-type heme, differentiating it from all previously characterized HOs. The crystal structure of Pden_1323 contains only a partial active site relative to HugZ, suggesting that it can accommodate a *c*-type hemopeptide substrate that provides its own histidine ligand. Activity assays of Pden_1323 with the hemopeptide substrate microperoxidase-11 (MP-11) show *c*-type heme degradation to free iron, CO, and a peptide-linked β- and/or δ-biliverdin. The structural and kinetic results together demonstrate Pden_1323 as a novel *c*-type heme oxygenase (CHO), indicating the ability of *P. denitrificans* to acquire iron from direct oxidative cleavage of *c*-type cytochrome fragments containing heme.

## Results

### Pden_1323 lacks PMP/PNP oxidase and HO activity

**Figure 3:**
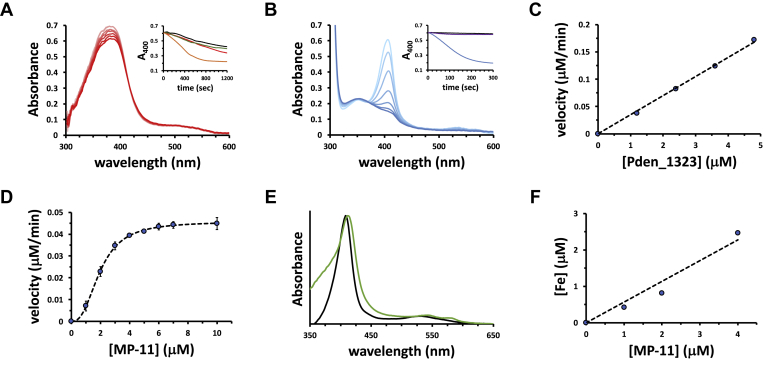
**Spectrophotometric assays demonstrating *c*-type heme oxygenase (CHO) activity of Pden_1323.***A*, UV–visible absorption spectra of the Pden_1323 reaction with 25 μM hemin and 10 mM ascorbate taken every 50 s for 5 min. *Inset*, time course of hemin degradation measured by the decrease in intensity of the Soret band in the presence (*red trace*) or absence (*black trace*) of Pden_1323. Reactions were also carried out in the presence of 1.8 mM imidazole both with (*orange trace*) and without (*green trace*) Pden_1323. *B*, UV–visible absorption spectra of the Pden_1323 reaction with 5.4 μM MP-11 and 10 mM ascorbate taken every 50 s for 5 min. *Inset*, time course of MP-11 degradation measured by the decrease in intensity of the Soret band in the presence (*blue trace*) or absence (*black trace*) of Pden_1323. A reaction was also performed in the presence of Pden_1323 but omitting the reductant ascorbate (*purple trace*). *C*, comparison of the rates of degradation of 9 μM MP-11 as a function of Pden_1323 concentration in the presence of 2 μM catalase. *D*, initial rate of the Pden_1323 reaction as a function of [MP-11] in the presence of 2 μM catalase. The *dashed line* shows best fit to the Hill equation using the kinetic parameters *k*_cat_ = 0.076 ± 0.0006 min^−1^, *K*_m_ = 2.0 ± 0.03 μM, and *n* = 2.6 ± 0.08. *E*, myoglobin assay showing the production of CO during the degradation of MP-11 in the presence of Pden_1323 (*green trace*) but not in the absence of enzyme (*black trace*). *F*, ferrozine assay showing Pden_1323-catalyzed production of free iron (relative to a no enzyme control) as a function of MP-11 concentration.

The codon-optimized version of *pden_1323* was synthesized for heterologous expression in *E. coli*. The purified enzyme was tested for its ability to oxidize PMP and pyridoxine 5′-phosphate (PNP), but no activity was observed with either substrate using FMN, FAD, F_420_, or 8-hydroxy-5-deazaflavin (F_o_). Pden_1323 was also tested for HO activity using hemin as the substrate. Again, no significant activity above the background level was detected using ascorbate as the reductant (Fig. 3*A*).

### Crystral structure of Pden_1323 and comparison to HugZ

The X-ray crystal structure of Pden_1323 was solved and found to form a dimeric split β-barrel similar to HugZ (Fig. 2) (25). However, comparison of the active sites of Pden_1323 and HugZ shows stark differences. While the two residues of HugZ that form salt bridging interactions with the carboxylates of heme, His163 and Arg166, are conserved in the structure of Pden_1323 (His64 and Arg92), the entire N-terminal α/β domain of HugZ is not present in the structure of Pden_1323 (25). Furthermore, the C-terminal loop that contains the coordinating heme ligand is not found in the Pden_1323 structure (Fig. 2) (25). Taken together, the lack of the N-terminal domain and C-terminal loop indicates that the presumed active site of Pden_1323 is only partially formed, which provides a rationale for Pden_1323's lack of HO activity with free heme. Given these structural characteristics, combined with the fact that *pden_1323* is found within a gene cluster that is likely involved in the transport of both heme and peptides, it was reasoned that the substrate of Pden_1323 may instead be a *c*-type hemopeptide.

### Substrate specificity and kinetics of Pden_1323

**Figure 4:**
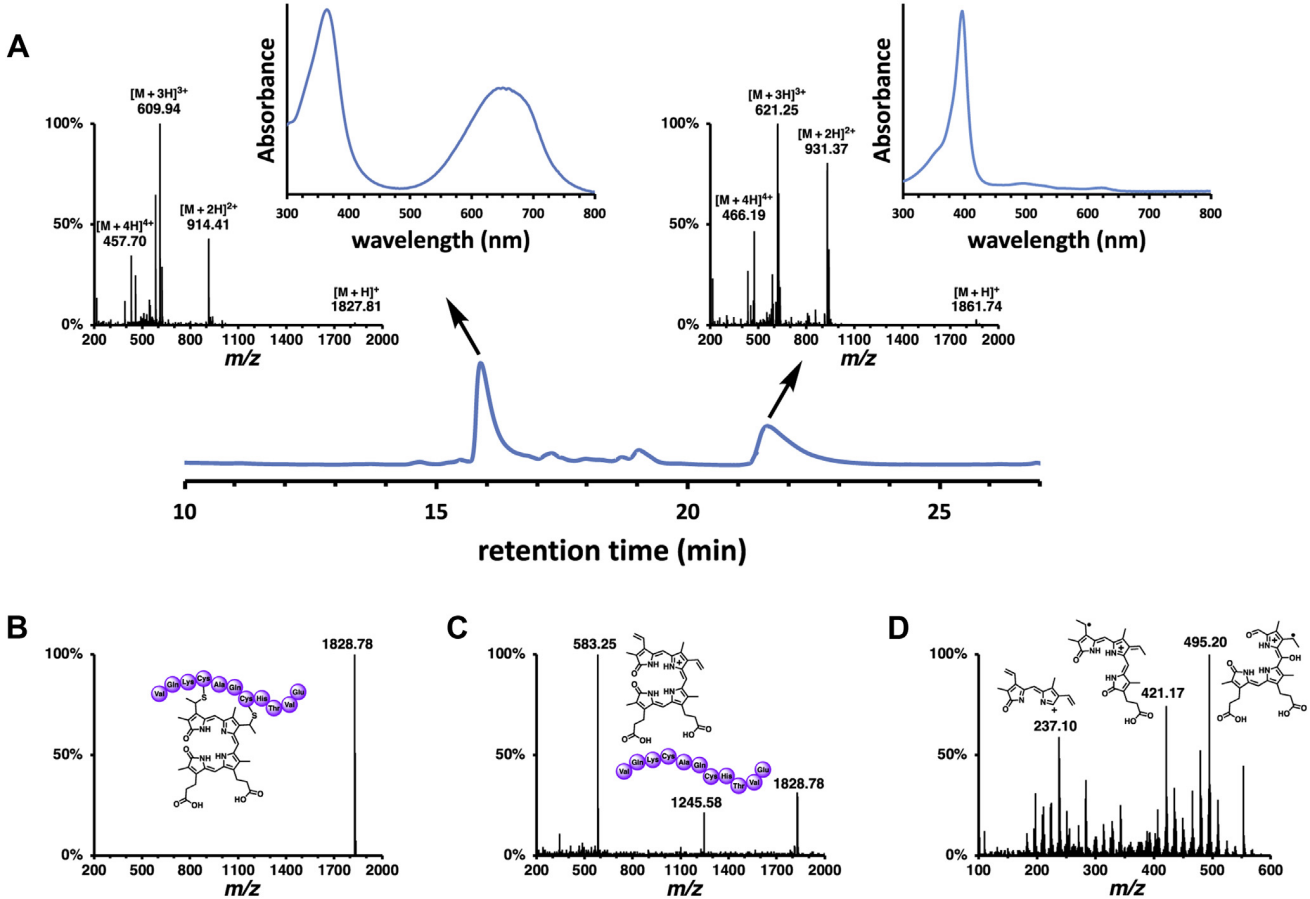
**HPLC and MS analyses confirming the product of the Pden_1323 reaction with MP-11 as a peptide-linked biliverdin.***A*, HPLC analysis of a Pden_1323 reaction mixture, showing MS and UV–visible absorption spectra of the reactant (21.6 min) and product (15.9 min) peaks. *B*, simplified MS/MS spectrum of the product of the Pden_1323 reaction with MP-11 using a linear range of collision energies from 10 to 30 eV. *C*, simplified MS/MS spectrum of the product of the Pden_1323 reaction with MP-11 using a linear range of collision energies from 60 to 90 eV. *D*, LC-MS/MS spectrum of the 583.25 *m/z* peak of the Pden_1323 reaction with MP-11.

When Pden_1323 was incubated with ascorbate and MP-11, an 11 amino acid heme-containing fragment of equine heart cytochrome *c*, a rapid decrease in the Soret band of MP-11 was observed (Fig. 3*B*) (34). The loss of the *c*-type heme chromophore was both enzyme and reductant-dependent (Fig. 3, *B* and *C*). In the presence of catalase, the Pden_1323-catalyzed degradation of MP-11 occurred with a specific activity of 3.9 nmol/min/mg and displayed sigmoidal kinetics with a turnover number (*k*_cat_) of 0.076 ± 0.0006 min^−1^, an apparent dissociation (Michaelis) constant (*K*_m_) of 2.0 ± 0.03 μM, and a Hill coefficient of 2.6 ± 0.08 (Fig. 3*D*). Both CO (Fig. 3*E*) and free iron (Fig. 3*F*) were detected as coproducts of the reaction (using myoglobin and ferrozine assays, respectively). High-performance liquid chromatography (HPLC) analysis of the Pden_1323 reaction mixture showed conversion of MP-11 to a product with a mass (theoretical [M + H]^+^ *m*/*z* = 1827.82) and UV–visible absorption spectrum that are consistent with a peptide-linked biliverdin (Fig. 4*A*). Tandem mass spectrometry (MS/MS) of the Pden_1323 reaction product showed that the 1827.82 ion could be cleaved into peptide (theoretical *m*/*z* = 1245.57) and bilin (theoretical *m*/*z* = 583.26) components by increasing the collision energy (Fig. 4, *B* and *C*). LC-MS/MS analysis of the bilin peak (Fig. 4*D*) yielded a fragmentation pattern that is distinct from that obtained with α-biliverdin (Fig. S1) and consistent with oxidized biliverdin fragments containing A-B-C (or B-A-D) rings (theoretical *m*/*z* = 421.20) and C-B-D (or A-D-C) rings (theoretical *m*/*z* = 495.20). Thus, the MS data suggest that the product of the reaction is a peptide linked β- and/or δ-biliverdin.

No Pden_1323-catalyzed heme degradation activity was observed when full-length cytochrome *c* was used as the substrate (Fig. S2). However, when cytochrome *c* was digested with trypsin to generate a short 9 amino acid hemopeptide (*i.e.*, MP-9), rapid loss of the Soret band was once again observed at a rate comparable to that of MP-11 (Fig. S3). The MP-9 sequence is missing the three N-terminal residues of MP-11 (VQK) and has a C-terminal lysine not present in the MP-11 sequence. This suggests that the exact sequence of the hemopeptide is not critical for recognition and cleavage by Pden_1323, though the coordinating histidine residue is present in both MP-11 and MP-9. Consistent with this observation, when hemin was incubated with a high concentration of imidazole (where ∼90% is expected to be coordinated given an estimated *K*_d_ ∼ 200 μM), Pden_1323 is now able to cleave it (Fig. 3*A*).

## Discussion

Taken together, the activity assays demonstrate that Pden_1323 is a novel *c*-type heme oxygenase (CHO) that shows specificity for the degradation of *c*-type hemopeptides over free *b*-type heme. The results of the MS experiments and myoglobin/ferrozine assays further indicate that Pden_1323 catalyzes the oxidative cleavage of *c*-type hemopeptides to a peptide-linked β- and/or δ-biliverdin with concomitant formation of CO and free iron. The inability of Pden_1323 to cleave full-length cytochrome *c* shows that the peptide linkage must be small enough so that the porphyrin macrocycle is not buried and is able to access the active site, while the cleavage of hemin at high imidazole concentration indicates that the histidine lower axial ligand (rather than the *c*-type thioether linkages or the peptide sequence) is the crucial factor enabling catalysis. This suggests that Pden_1323 may bind a range of hemopeptide substrates in a way that creates a HugZ-like active site, with the histidine residue provided by the peptide substrate coordinating and activating the heme iron. After substrate binding, the mechanism of Pden_1323-catalyzed *c*-type heme degradation is expected to proceed analogously to that of HugZ (25). The exact physiological role of Pden_1323 in the soil microbe *P. denitrificans* has yet to be established. It may be involved in acquiring iron from environmental hemopeptides (*e.g.*, from degraded extracellular cytochromes). However, given the large number of *c*-type cytochromes utilized by this metabolically versatile bacterium, Pden_1323 may also play an important role in general cell maintenance by recycling iron from damaged cytochromes (30).

The structural comparison of HugZ and Pden_1323 suggests that the change in substrate specificity from free heme *b* to a *c*-type hemopeptide involves the loss of the C-terminal loop containing the coordinating heme ligand and an opening of the active site to accommodate the larger substrate *via* loss of the N-terminal α/β domain (25). Similar structural features are also observed in the heme storage protein HutZ from *Vibrio cholerae* and the heme utilization protein HupZ from Group A *streptococcus* (GAS) (35, 36). HutZ is required for optimal heme utilization in *V. cholerae*, although no HO activity was initially observed with hemin (35). However, subsequent work by Uchida and coworkers suggests that HutZ functions as a HO at pH values below 8.0, producing the typical oxyferrous heme, meso-hydroxyheme, and verdoheme intermediates with regiospecificity similar to HugZ (37, 38). While HutZ lacks the N-terminal α/β domain, it retains the C-terminal loop containing the conserved histidine lower axial ligand, which is important for its heme degradation activity (39). In contrast, HupZ lacks both the N-terminal α/β domain and C-terminal loop and thus more closely resembles Pden_1323 (36). Interestingly, HupZ was shown to copurify with heme when it was overexpressed in *E. coli* grown in the presence of 5-aminolevulinic acid and iron and demonstrated modest hemin degrading activity (36). However, this enzyme was expressed as a C-terminal His-tagged fusion protein, and it is possible that the affinity tag enhanced the activity of HupZ with free heme (36). Indeed, during the revision of this article, Traore *et al*. (40) published a paper reinvestigating GAS HupZ and found that the weak heme-degradation activity (and an observed oligomerization state change) is associated with heme binding to the C-terminal His-tag. Thus, it is possible that HupZ and other heme storage/utilization proteins may also show preference for the degradation of heme *c*.

In developed countries, dietary heme accounts for approximately two-thirds of an average person's iron stores, though heme *b* present in consumed hemoglobin and myoglobin is considered to be the largest iron source (41). However, acid or enzymatic hydrolysis of cytochromes in the gastrointestinal tract can result in soluble hemopeptides, as well as polypeptides known to increase the solubility of free heme (42). Enterocytes utilize heme carrier protein 1 (HCP1) to move free heme into the cell from the intestinal lumen (43). To the best of the authors' knowledge, HCP1 has not been shown to transport hemopeptides. Therefore, enteric bacteria with an appropriate Opp system and a homolog of Pden_1323 could gain a competitive advantage within the gut microbiome by exploiting this alternative iron source resulting from cytochrome degradation, with little competition for hemopeptide substrates from enterocytes. In the case of pathogenic bacteria (such as the aforementioned Group A *streptococcus*) that rely on this pathway for iron acquisition, inhibiting CHOs could lead to greatly reduced iron availability for the pathogen while leaving enterocytes relatively unaffected. Further mechanistic studies of Pden_1323, and its possible inhibition mechanisms, are needed to investigate this hypothesis.

In summary, a novel HO from *P. denitrificans* with specificity for the degradation of *c*-type hemopeptides was identified and structurally characterized. The oxygenation reaction with MP-11 rapidly yields free iron, CO, and a peptide-linked β- and/or δ-biliverdin as products. The structural features conferring specificity for heme *c* are also observed in a number of heme storage/utilization proteins from pathogenic bacteria, suggesting that these organisms may also target the *c*-type heme pool as a source of iron to sustain infections.

## Experimental procedures

### Materials

Biliverdin hydrochloride, hemin chloride, imidazole, and sodium ascorbate were acquired from Alfa Aesar. MP-11, cytochrome *c*, catalase, myoglobin, and FMN were purchased from Sigma-Aldrich. FAD was obtained from Tokyo Chemical Industry (TCI). F_420_ and F_o_ were generous gifts from Dr Kayunta Johnson-Winters (University of Texas, Arlington). The FerroZine iron reagent and neocuproine hemihydrate used for iron determination were from Acros Organics. LB media was from Becton, Dickinson and Company (BD), while buffer components were from VWR Chemicals BDH. Primers were ordered from Sigma-Aldrich and the synthesized *pden_1323* gene was purchased from GeneArt. Phusion High-Fidelity PCR Kit, T4 DNA ligase, and restriction endonucleases were from New England Biolabs. Gel extraction, PCR cleanup, and mini prep kits were purchased from Omega Bio-Tek. Profinity IMAC resin was obtained from Bio-Rad.

### Vector construction

The *pden_1323* gene from *P. denitrificans* PD1222 was codon-optimized for expression in *E. coli* with NdeI and XhoI restriction sites included at the 5′ and 3′ ends, respectively. The vector containing *pden_1323*, as well as an empty pET-28b(+) vector, was digested with these restriction enzymes. The *pden_1323* product was purified *via* a Lonza FlashGel DNA Cassette and recovered using an Omega Bio-tek E.Z.N.A. Gel Extraction Kit, while the linearized pET-28b(+) vector was purified using an Omega Bio-tek E.Z.N.A. Cycle Pure Kit. The *pden_1323* insert and the digested pET-28b(+) vector were then ligated together using T4 DNA ligase using the manufacturer's protocol. The ligated mixture was then transformed into *E. coli* TOP10 cells and plated on Luria–Bertani (LB) agar plates containing 50 μg/ml kanamycin. Colonies were picked, grown in liquid LB medium, and plasmid preparations were made using the Omega Bio-tek E.Z.N.A. Plasmid Mini Kit.

### Protein expression and purification

The pET-28b(+):*pden_1323* expression vector was transformed into *E. coli* BL21(DE3) cells. The transformed expression host was grown in overnight cultures (2 ml of LB with 50 μg/ml kanamycin) that were used to inoculate 6 × 1 L of LB (50 mg/L kanamycin). The cultures were incubated with shaking at 37 °C until the OD_600_ reached 0.6. The cultures were then induced with isopropyl β-d-1-thiogalactopyranoside (IPTG) at a final concentration of 0.1 mM and incubated for an additional 4 h. The cells were centrifuged at 15,900*g* for 45 min. The supernatant was decanted and the cells were resuspended in 50 mM sodium phosphate buffer (pH 8.0) containing 300 mM NaCl and 5 mM imidazole. The cells were then sonicated and centrifuged at 15,900*g* for 1 h. The supernatant was loaded onto a Bio-Rad Econo-Pac column loaded with nickel-charged Profinity IMAC resin. The column was washed with 50 mM sodium phosphate buffer (pH 8.0) containing 300 mM NaCl and 5 mM imidazole. The protein was then eluted from the column by adding 50 mM sodium phosphate buffer (pH 8.0) containing 300 mM NaCl and 500 mM imidazole in 1 ml fractions. The protein-containing fractions were combined and spin-concentrated/buffer exchanged into 100 mM Tris buffer (pH 8.0) followed by incubation with thrombin (1:1000 [w:w]) overnight at room temperature to remove the N-terminal His-tag. After incubation, the cleaved protein was passed through a benzamidine/Ni-NTA column. The eluted protein was then spin-concentrated/buffer exchanged into 100 mM Tris buffer (pH 8.0) containing 15% glycerol and separated into 100 μl aliquots.

For crystallographic studies, the transformed cultures were induced with 0.4 mM IPTG and grown for an additional 3 h. Cells were harvested *via* centrifugation (15,900*g* for 10 min), and the pelleted cells were stored frozen at –80 °C. The frozen cells were thawed and resuspended in lysis buffer (50 mM Tris (pH 7.5) buffer with 10% (v/v) glycerol and 500 mM NaCl) for sonication. The lysed cells were centrifuged (15,900*g* for 20 min), and the supernatant was incubated with Ni-NTA agarose resin (Qiagen) at 4 °C for 30 min and loaded into a column. The column was washed with 15 column volumes of 20 mM imidazole in lysis buffer before Pden_1323 was eluted with 500 mM imidazole in lysis buffer. Pden_1323 was desalted and buffer exchanged into a thrombin cleavage buffer (20 mM Tris (pH 8.0) containing 25 mM CaCl_2_ and 100 mM NaCl) followed by incubation with thrombin (1:1000 [w:w]) overnight at 25 °C to remove the N-terminal His-tag. After incubation, the cleaved protein was passed through a benzamidine/Ni-NTA column. Pden_1323 was further purified over a gel filtration column (Superdex 200, GE Healthcare Life Sciences) equilibrated with 50 mM Tris buffer (pH 7.5) containing 5% (v/v) glycerol and 150 mM NaCl. An Amicon Stirred Cell protein concentrator was used to exchange the protein into 10 mM Tris (pH 7.5) buffer containing 5% (v/v) glycerol and 25 mM NaCl and achieve a final concentration of 12 mg/ml. Aliquots were flash-frozen in liquid nitrogen and stored at –80 °C until further use.

### Heme oxygenase activity assays

The CHO activity of Pden_1323 was examined by incubating 2.8 μM enzyme and 5.4 μM MP-11 with 10 mM sodium ascorbate in a cuvette containing 20 mM potassium phosphate buffer (pH 7.6). UV–visible absorption spectra were recorded every 2 seconds for 5 min using a NanoDrop 2000c (Thermo Fisher). Control reactions were performed with no enzyme or reductant and with MP-11 replaced by 25 μM hemin (with or without 1.8 mM imidazole), 5.4 μM cytochrome *c*, or 1.7 μM MP-9. MP-9 was obtained by incubating 5 mg of cytochrome *c* with 5 mg of trypsin in 1 ml of 20 mM potassium phosphate buffer (pH 7.6) overnight at room temperature. The MP-9 hemopeptide was then purified by HPLC using an Agilent 1260 Infinity Quaternary LC System equipped with a Diode Array Detector VL+, an analytical scale 1260 Fraction Collector, and an Agilent Poroshell 120 EC-C18 (4.6 × 150 mm, 2.7 μm) column. The mobile phase consisted of water with 0.5% formic acid (solvent A) and acetonitrile with 0.5% formic acid (solvent B). The chromatographic method used to isolate MP-9 consisted of a linear gradient of 0–100% solvent B over 30 min, with a flow rate of 1.0 ml/min and detection at 400 nm.

Another reaction containing 135 μM MP-11, 100 μM Pden_1323, 5 mM sodium ascorbate, and 8 μM catalase in 200 ml of 20 mM potassium phosphate buffer (pH 7.6) was quenched after 30 min with 200 ml methanol and subjected to HPLC analysis. The same LC system and mobile phases as above were utilized with the following gradient: 0% B for 2 min, linear gradient to 20% B over 3 min, 20% B for 5 min, linear gradient to 25% B over 5 min, 25% B for 5 min, linear gradient to 30% B over 5 min, linear gradient to 100% B over 2 min, and 100% B for 3 min. A flow rate of 1.0 ml/min was used, and the trace was acquired using detection at 280 nm. The reactant and product peaks were analyzed with UV–visible absorption spectroscopy (*via* the in-line diode array detector) and subjected to mass spectrometry (MS) analysis as detailed below.

The dependence of the initial rate of the CHO reaction on the concentration of enzyme was determined by incubating 9 μM MP-11, 10 mM sodium ascorbate, 2 μM catalase, and 1.2–4.8 μM Pden_1323 in a cuvette containing 20 mM potassium phosphate buffer (pH 7.6). The dependence of the initial rate of the CHO reaction on the concentration of MP-11 was determined by incubating 0.6 μM enzyme, 10 mM sodium ascorbate, 2 μM catalase, and 1–10 μM MP-11 in a cuvette containing 20 mM potassium phosphate buffer (pH 7.6) in a total volume of 400 μl. The reactions were monitored at 406 nm and were performed in triplicate using an Agilent 8453 UV-visible Spectrophotometer. The latter data were then fit to the Hill equation,

$$v=\frac{k_{cat}\left[\text{Pden}\_1323\right]{\left[MP\;-\;11\right]}^{n}}{{K_{\text{m}}}^{n}\;+\;{\left[MP\;-\;11\right]}^{n}}$$

to obtain estimates for the turnover number (*k*_cat_), apparent dissociation (Michaelis) constant (*K*_m_), and Hill coefficient (*n*) of Pden_1323/MP-11.

### CO detection assays

To detect the production of CO in the Pden_1323 reaction, a mixture of 50 μM Pden_1323, 50 μM MP-11, 10 mM sodium ascorbate, and 2 μM catalase was placed in a sealed vial containing 20 mM potassium phosphate buffer (pH 7.6). A control reaction was also performed without Pden_1323. After 1 h, 100 μl of both the Pden_1323 and control reactions were mixed with 300 μl of an anaerobic myoglobin solution (10 μM final concentration) in an anaerobic chamber. UV–visible absorption spectra were then recorded using a NanoDrop One^C^ (Thermo Fisher).

### Iron determination assay

To confirm the production of free iron in the Pden_1323 reaction, 1.2 μM Pden_1323 was incubated with 10 mM sodium ascorbate, 2–10 μM MP-11, 2 μM catalase, and 40 μl ferrozine reagent B (2 M ascorbic acid, 5 M ammonium acetate, 6.5 mM ferrozine, 13.1 mM neocuproine) in a cuvette containing a total of 400 μl of 20 mM potassium phosphate buffer (pH 7.6). A control reaction was also performed without enzyme. The reactions were monitored at 562 nm using an Agilent 8453 UV-visible Spectrophotometer.

### Mass spectrometry analysis

MS analysis of the Pden_1323 reaction mixtures was performed at the Auburn University Chemistry and Biochemistry Mass Spectrometry Center on an ultra-performance LC system (ACQUITY, Waters Corp) coupled with a quadrupole time-of-flight mass spectrometer (Q-TOF Premier, Waters Corp) with electrospray ionization (ESI) in positive mode using MassLynx software (v4.1). Injections of the samples were made directly into the mass spectrometer or onto a C4 column (Aeris 3.6 μm C4 200 Å, 50 × 2.1 mm, Phenomenex) with a 300 μl/min flow rate of mobile phase solution A (0.1% formic acid in 95% water and 5% acetonitrile) and solution B (0.1% formic acid in 95% acetonitrile and 5% water) using the following gradient: 0% B for 2 min, linear gradient to 100% B over 11 min, 100% B for 1 min, linear gradient to 0% B over 1 min, and 0% B for 3 min. The column temperature was held at 40 °C and the UV detector monitored absorption at 420 nm. The capillary voltage was set at 3.1 kV, the sample cone voltage was 15 V, and the extraction cone was 4.0 V. The source and desolvation temperature were maintained at 95 and 300 °C, respectively, with the desolvation gas flow set to 600 L/h. The MS scan was 0.5 s long from 50 to 2000 *m*/*z* with a 0.02 s interscan delay using the centroid data format. Tandem mass spectra were taken for selected masses with a 0.5 s long scan and 0.025 s interscan delay over the same range. The collision energy varied as described in the text. The lock mass was used to correct instrument accuracy with a 2 ng/μl solution of leucine enkephalin.

### Crystallization, data processing, and structure determination

Three sparse matrix screens (The JCSG Core IV, The JCSG + and The Protein Complex Suites, Qiagen) were used to determine crystallization conditions for Pden_1323. Several conditions produced crystals, and optimization plates were set up for the following condition: 14% (w/v) glycerol, 18 to 25% (w/v) PEG 4000, 0.3 M sodium acetate, and 0.1 M Tris (pH 8.8). Crystals grew overnight *via* the sitting-drop vapor diffusion method. Each drop consisted of 1 μl of Pden_1323 (12 mg/ml) and 2 μl of crystallization buffer. Crystals were cryoprotected with 2 μl of glycerol in 8 μl mother liquor for 5 min, then flash frozen in liquid nitrogen prior to data collection.

Data for Pden_1323 were collected on the Advanced Photon Source Beamline 23-ID-B and subsequently processed with HKL-3000 (Table S1) (44). The structure of Pden_1323 was determined by molecular replacement with the Atu2129 monomer (PDB ID code: 3DNH) as a search model using Phaser (45, 46). The model was initially refined with ARP/wARP and then built and refined through several cycles with Coot and Refmac (47, 48, 49). The final structure was optimized using the PDB_REDO server (50).

## Data availability

The coordinates and structure factors for the crystal structure of Pden_1323 have been deposited in the Protein Data Bank under PDB ID code 6VNA.

## Supporting information

This article contains supporting information.

## Conflict of interest

The authors declare that they have no conflicts of interest with the contents of this article.
