# Supplementary material for: A noncanonical heme oxygenase specific for the degradation of c-type heme
Source: J Biol Chem. 2021 Apr 17;296:100666. doi: 10.1016/j.jbc.2021.100666 (PMC8131568; doi:10.1016/j.jbc.2021.100666)
Supplement: Figures S1 to S3 and Table S1 [file mmc1.pdf]

Supplemental Materials

A noncanonical heme oxygenase specific for the degradation of *c*-type heme

Shuxin Li[1], Eta A. Isiorho[1,2], Victoria L. Owens[1], Patrick H. Donnan[1], Chidinma L. Odili[1], and Steven O. Mansoorabadi[1]*

[1]Department of Chemistry and Biochemistry, Auburn University, Auburn, Alabama 36849, USA

[2]Present address: Advanced Science Research Center, City University of New York, New York, NY 10031, USA

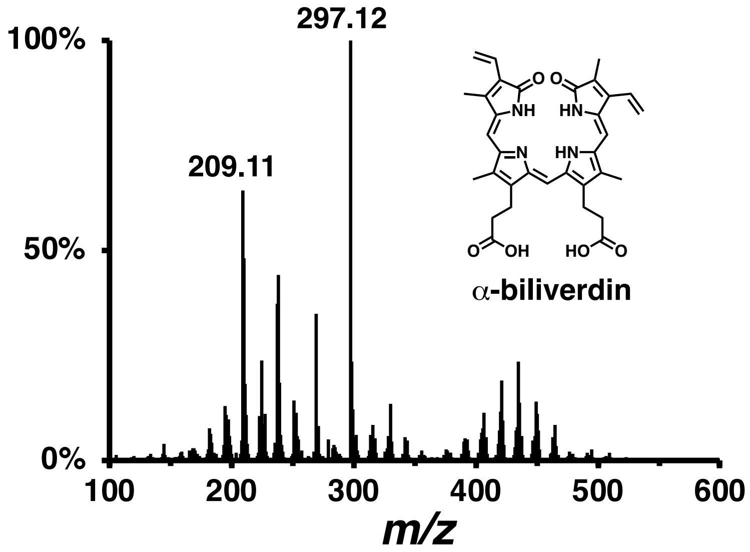

Figure S1. LC-MS/MS spectrum of a biliverdin IXα standard.

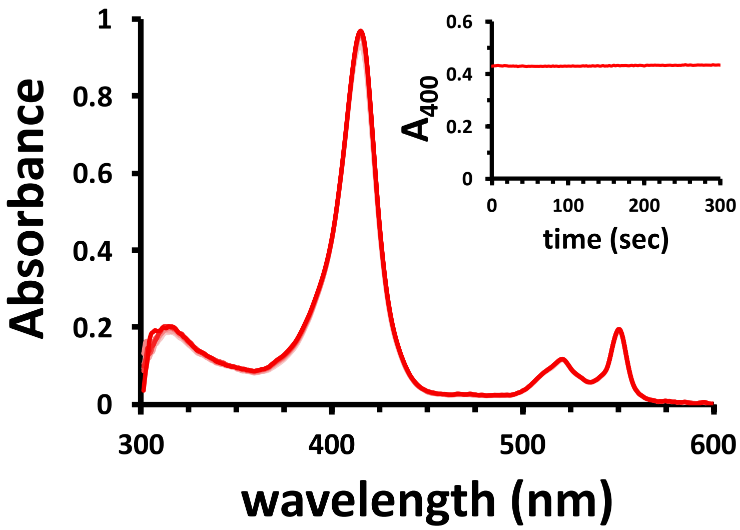

Figure S2. Spectrophotometric assays of Pden_1323 with full-length cytochrome *c* as substrate. UV-visible absorption spectra of the Pden_1323 reaction with 5.4 µM cytochrome *c* and 10 mM ascorbate taken every 50 seconds for 5 minutes. Inset: Time course of the reaction followed by monitoring the intensity of the Soret band.

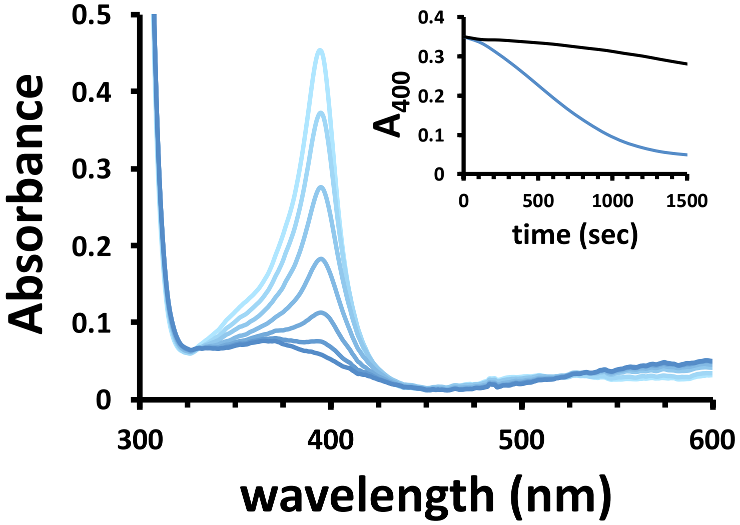

Figure S3. Spectrophotometric assays of Pden_1323 with MP-9 as substrate. UV-visible absorption spectra of the Pden_1323 reaction with 1.7 μM MP-9 and 10 mM ascorbate taken every 4 minutes. Inset: Time course of MP-9 degradation measured by the decrease in intensity of the Soret band in the presence (blue trace) or absence (black trace) of Pden_1323.

Table S1. Data collection and refinement statistics (molecular replacement).

	**Pden_1323**
**Data collection**	
Space group	C2
Cell dimensions	
a, *b*, *c* (Å)	72.84, 117.04, 59.38
α, β, γ (°)	90, 99.38, 90
Resolution (Å)	58.66 – 2.20 (2.27 – 2.20)
R_{merge}	0.037(0.378)
Completeness (%)	99.6(99.3)
Redundancy	2.0(1.9)
**Refinement**	
Resolution (Å)	2.20
No. reflections	24905
R_{work} / R_{free}	0.212/0.232
No. atoms	
Protein	6562
Ligand/ion	
Water	47
B-factors	
Protein	69
Ligand/ion	
Water	47
R.m.s. deviations	
Bond lengths (Å)	0.0067
Bond angles (°)	1.181
